# Associations between Sjögren Syndrome, Sociodemographic Factors, Comorbid Conditions, and Scleritis in a Taiwanese Population-Based Study

**DOI:** 10.3390/jpm12010105

**Published:** 2022-01-14

**Authors:** Ren-Long Jan, Chung-Han Ho, Jhi-Joung Wang, Sung-Huei Tseng, Yuh-Shin Chang

**Affiliations:** 1 Department of Pediatrics, Chi Mei Medical Center, Liouying, Tainan 736, Taiwan; renlongjan@gmail.com; 2 Graduate Institute of Medical Sciences, College of Health Sciences, Chang Jung Christian University, Tainan 711, Taiwan; 3 Department of Medical Research, Chi Mei Medical Center, Tainan 710, Taiwan; ho.c.hank@gmail.com (C.-H.H.); 400002@mail.chimei.org.tw (J.-J.W.); 4 Department of Hospital and Health Care Administration, Chia Nan University of Pharmacy and Science, Tainan 717, Taiwan; 5 Department of Anesthesiology, Chi Mei Medical Center, Tainan 710, Taiwan; 6 Department of Ophthalmology, National Cheng Kung University Hospital, College of Medicine, National Cheng Kung University, Tainan 701, Taiwan; shtseng1@gmail.com; 7 Department of Ophthalmology, Chi Mei Medical Center, Tainan 710, Taiwan

**Keywords:** scleritis, Sjögren syndrome, case–control study, Taiwan Longitudinal Health Insurance Database

## Abstract

This nationwide, population-based, retrospective, matched case–control study included 111,960 newly diagnosed patients with scleritis who were identified by the International Classification of Diseases, Ninth Revision, Clinical Modification code 379.0, selected from the Taiwan National Health Insurance Research Database. Demographic characteristics, Sjögren syndrome, and comorbid conditions within 1 year before the scleritis diagnosis were examined using univariate logistic regression analyses, and a paired *t*-test was used for continuous variables. Adjusted logistic regression was used to compare the prognosis odds ratio (OR) of the patients with scleritis with the controls. After adjustment for confounders, patients with Sjögren syndrome were remarkably more likely to have scleritis than the controls (OR = 33.53, 95% confidence interval (CI) = 27.43–40.97, *p* < 0.001). Other conditions found to have increased odds of scleritis included post ocular pterygium, glaucoma, and scleral surgery (OR  =  4.01, 95% CI  =  3.64–4.43; OR  =  3.16, 95% CI  =  2.24–4.47; OR  =  6.83, 95% CI  =  5.34–8.74, respectively); systemic infections, such as syphilis, tuberculosis, and a human herpes viral infection (OR  =  4.01, 95% CI  =  2.93–5.50; OR  =  2.24, 95% CI  =  1.94–2.58; OR  =  8.54, 95% CI  =  8.07–9.03, respectively); and systemic diseases, such as rheumatoid arthritis, granulomatous vasculitis, systemic lupus erythematosus, ankylosing spondylitis, and gout (OR  =  2.93, 95% CI  =  2.66–3.23; OR  =  7.37, 95% CI  =  3.91–13.88; OR  =  3.18, 95% CI  =  2.63–3.85; OR  =  5.57, 95% CI  =  4.99–6.22; OR  =  2.84, 95% CI  =  2.72–2.96, respectively). The results strongly support an association between Sjögren syndrome, post ocular surgery, systemic infection disease, systemic autoimmune disease, and scleritis.

## 1. Introduction

Scleritis, a severe ocular inflammatory condition, is characteristically painful and can cause significant visual impairment [1]. The sclera, the outer covering of the eye, has unique structural and vascular characteristics that are vulnerable to inflammatory conditions. The structure of the sclera is composed of a scaffold of collagen, protein fibrils, glycoproteins, and proteoglycans. In particular, the relatively avascular nature of the sclera decreases circulation and antigens or harmful substance removal, that can lead to intense inflammatory reactions [2].

Scleritis tends to affect patients in middle age, with the median age at diagnosis being between 47 and 60 years. It is more predominant in the female sex, with women comprising 60% to 74% of patients [1,3,4,5,6]. There are multiple etiologies for scleritis, including infectious and non-infectious conditions. Surgically induced infectious scleritis has been associated with pterygium excision followed by scleral buckle surgery [7]. Infectious scleritis can also be caused by viruses, bacteria, and fungi infections and are reported to occur in 4–10% of all scleritis cases [1,8]. Herpetic scleritis is the most common cause of infectious scleritis and is more likely to cause visual impairment [9]. Up to 50% of patients with scleritis have a systemic autoimmune condition, most commonly rheumatoid arthritis, followed by systemic vasculitis diseases [1,10,11,12,13]. In these autoimmune conditions, rheumatoid arthritis typically results in swollen and tender joints and Sjögren syndrome mostly affects the lacrimal and salivary glands and other organs. Sjögren’s syndrome can occur independently or follow other autoimmune disorders, such as rheumatoid arthritis or systemic lupus erythematosus.

Scleritis, a sight-threatening condition, may be linked to systemic immune-mediated inflammatory disease, which is the result of aberrant immune responses and often needs systemic immunosuppression to avoid aggravation [14]. A major problem to approaching evidence-based management of scleritis is the limited population-based epidemiologic data on the strength of associations of scleritis and systemic inflammatory disease [14]. It is hence necessary to investigate the association between scleritis and immune-mediated disease to increase the awareness of scleritis as one of the causes of ocular problems in patients with inflammatory diseases.

The purpose of the current study was to use a health care claims database containing records for more than 110,000 patients with scleritis and controls matched by age and sex, to investigate the association between Sjögren syndrome, sociodemographic factors, various comorbid conditions, such as post ocular surgery, systemic infections, systemic autoimmune diseases, and scleritis, which may help to elucidate the pathophysiologic features of scleritis.

## 2. Materials and Methods

### 2.1. Database

The data for our case–control study was taken from the National Health Insurance Research Database (NHIRD), provided by the National Health Research Institute (NHRI), Taiwan. The NHIRD provides encrypted patient identification numbers together with information on patient demographics, such as age, sex, residential area, and dates of admission and discharge. It also incorporates the International Classification of Diseases, Ninth Revision, Clinical Modification (ICD-9-CM) codes, which records procedures, diagnoses, and prescription items, as well as costs covered by the NHRI. The present research was exempt from review by the Institutional Review Board of the Chi Mei Medical Centre.

### 2.2. Selection of Patients and Variables

**Figure 1:**
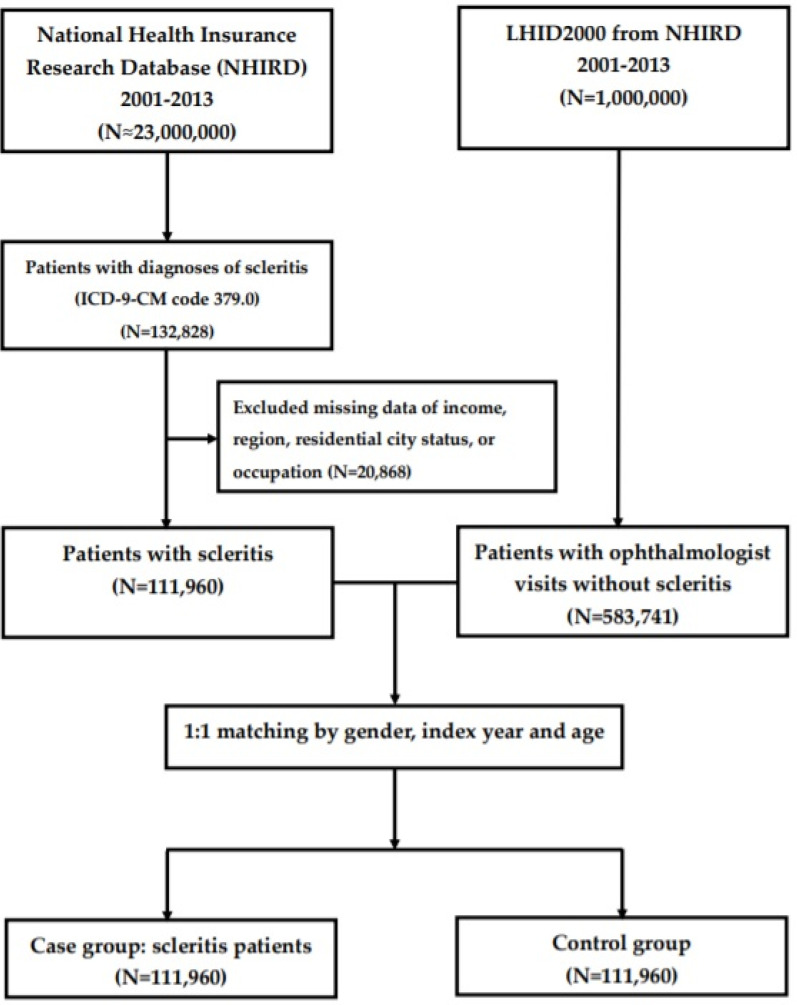
Flowchart demonstrating the enrolment of patients with scleritis and the control patients.

This population-based case–control study enrolled a newly diagnosed scleritis group and a matched non-scleritis control group. Data was collected from 1 January 2001 to 31 December 2013. A flowchart of our study is shown in Figure 1. Initially, 132,828 patients with a diagnosis of scleritis (ICD-9 cm code 379.0) were included in the study. From these, a total of 111,960 patients with newly diagnosed scleritis were enrolled after we excluded patients with missing demographic data on income, region, residential city status, or occupation. Therefore, 111,960 patients with a diagnosis of scleritis taken from the NHIRD were finally included after matching with the controls in the study.

For each patient with scleritis, one non-scleritis control was randomly chosen from the Longitudinal Health Insurance Database 2000 (LHID 2000), which is a subset of the NHIRD and contains the overall claims data for 1 million beneficiaries for the year 2000. Initially, we included 583,741 subjects who had reported at least 1 ophthalmology visit and did not have a scleritis diagnosis before the index date from the 1 million subjects recorded in the LHID 2000, after excluding patients with missing sex or demographic data. Controls (*n* = 111,960) were matched to the patients with scleritis via propensity scores by age (±30 days), sex, and index date, which was defined as the first day of diagnosis with scleritis. Each participant in both groups were tracked and the demographic data of each participant was recorded from the index date until the end of 2013 or death, whichever was earlier. To determine the medical comorbidities for scleritis, data regarding comorbid conditions, such as post-pterygium removal (order codes 87212C, 87213C, 85203C, and 85204C), post- glaucoma surgery (order codes 85805C, 85806C, and 85823B), post-scleral buckle replacement (order codes 85601C, 85604B to 85611B, 86406B, 86411B, and 86413B), syphilis (ICD-9-CM codes 090–097), tuberculosis (ICD-9-CM code 010–018), human herpes viral infection (varicella (ICD-9-CM codes 052.0–052.9), herpes zoster (ICD-9-CM codes 053–053.9), herpes simplex (ICD-9-CM codes 054–054.9 and 771.2), Epstein Barr virus (ICD-9-CM code 075), and cytomegaloviral disease (ICD-9-CM code 078.5)), Sjögren syndrome (ICD-9-CM code 710.2), rheumatic arthritis (ICD-9-CM code 714), granulomatous vasculitis (sarcoidosis (ICD-9-CM code 135), Wegener’s granulomatosis (ICD-9-CM code 446.4), polyarteritis nodosa (ICD-9-CM code 446.0), and giant cell arteritis (ICD-9-CM code 446.5)), systemic lupus erythematosus (ICD-9-CM codes 710.0 and 695.4), ankylosing spondylitis (ICD-9-CM code 720), and gout (ICD-9-CM codes 274 and V77.5) were collected. These comorbidities were identified based on an ICD-9-CM code being recorded within 1 year before the index date and ascertained by 3 or more ambulatory care claims or admittance as an inpatient.

### 2.3. Statistical Analysis

All statistical analyses were performed using SAS 9.4 for Windows (SAS Institute, Inc., Cary, NC, USA). McNemar’s test was used to analyse the demographic characteristics, such as age group, sex, income, geographic region, residential city status, and occupation. A paired *t*-test was utilized to calculate the continuous variables. In addition, comorbid conditions, mentioned above, were compared between the patients with scleritis and the controls using the McNemar’s test. Odds ratios (ORs), obtained by univariate logistic regression analyses and a multivariable logistic regression model (conditional on age, sex, and index date), were constructed to compute the adjusted OR for various comorbidities with a diagnosis of scleritis. The independent variables, including sociodemographic factors (income, geographic region, residential city status, and occupation) and all of the above-mentioned medical conditions of interest (post-pterygium removal, post-glaucoma surgery, post-scleral buckle replacement, syphilis, tuberculosis, human herpes viral infection (varicella, herpes zoster, herpes simplex, Epstein–Barr virus, and cytomegaloviral disease), Sjögren syndrome, rheumatic arthritis, granulomatous vasculitis (sarcoidosis, Wegener’s granulomatosis, polyarteritis nodosa, and giant cell arteritis), systemic lupus erythematosus, ankylosing spondylitis, and gout) were adjusted for in the regression model. The level of significance was set at *p* < 0.05.

## 3. Results

### 3.1. Demographic Data—Sociodemographic Factors

After ineligible patients were excluded, 111,960 patients with scleritis and 111,960 age- and sex-matched controls who had used medical care services covered by the NHRI between 2001 and 2013 were analysed. The mean ages of both the patients with scleritis and the controls were 42.92 years (standard deviation (SD) 15.33) (Table 1). Of the 111,960 patients with scleritis, 12,080 (10.79%) were younger than 25 years, 26,700 (23.85%) were aged 25–34 years, 26,776 (23.92%) were aged 35–44 years, 22,165 (19.80%) were aged 45–54 years, 13,707 (12.24%) were aged 55–64 years, and 10,532 (9.41%) were aged 65 years or over. Among the 111,960 patients with scleritis, 48,317 (43.16%) were men and 63,643 (56.84%) were women (Table 1). The incomes of the patients with scleritis were significantly different from the controls. The most common approximate income of the patients with scleritis was lower than 30,000 New Taiwan dollars (NT$) (57,986; 51.79%), followed by between NT$ 30,000 and 60,000 (41,226; 36.86%), between NT$ 60,000 and 90,000 (9458; 8.45%), and higher than NT$ 90,000 (3251; 2.90%) (*p* < 0.0001) (Table 1). With regard to geographic distribution, the most common region of residence of the patients diagnosed with scleritis was Northern Taiwan (61,637; 55.05%), followed by the southern (28,767; 25.69%), central (13,787; 12.31%), and eastern regions (7769; 6.94%), with a significant difference from the controls (*p* < 0.0001). Most of the patients with scleritis resided in a metropolis city (79,400; 70.92%) compared with those in a satellite city (6996; 6.25%) and rural areas (25,564; 22.83%), with a significant difference from the controls (*p* < 0.0001) (Table 1). With regard to occupation classification, a significant difference in the distribution was found between the 2 groups, with over half of the 111,960 patients with scleritis being public servants, including military, civil, or teaching staff (71,669; 64.01%), the remaining patients were farmers (7423; 6.63%) and fishermen (1585; 1.42%) (*p* < 0.0001) (Table 1).

### 3.2. Demographic Data—Comorbid Conditions

The patients with scleritis exhibited a significantly higher prevalence of needing eye wall surgery including pterygium removal (2513; 2.24%; *p* < 0.0001), scleral flap creation during glaucoma surgery (159; 0.14%; *p* < 0.0001), and scleral buckle replacement (568; 0.51%; *p* < 0.0001) than the controls (Table 1). A significantly higher prevalence of systemic infections in patients with scleritis, including syphilis (294; 0.26%; *p* < 0.0001), tuberculosis (1005; 0.90%; *p* < 0.0001), and human herpes viral infections (14,026; 12.53%; *p* < 0.0001) compared with the controls was noted (Table 1). There was evidence of significant differences regarding systemic diseases such as Sjögren syndrome (4588; 4.10%; *p* < 0.0001), rheumatoid arthritis (3298; 2.95%; *p* < 0.0001), granulomatous vasculitis (164; 0.15%; *p* < 0.0001), systemic lupus erythematosus (1009; 0.90%; *p* < 0.0001), ankylosing spondylitis (3012; 2.69%; *p* < 0.0001), and gout (11,313; 10.10%; *p* < 0.0001) between the patients with scleritis and the controls (Table 1).

### 3.3. Associated Risk Factors—Sociodemographic Factors

Sociodemographic factors, including income, geographic region, residential city status, and occupation of the patients with scleritis and the controls were examined using univariate logistic regression analyses and a multiple logistic regression model with adjustments for age, sex, sociodemographic factors, and comorbidities. Patients whose income was >NT$ 30,000 had increased odds of developing scleritis, relative to those with an income <NT$ 30,000, and this continued to be a significant risk factor for scleritis after adjustment for other confounders (Table 2). Regarding geographic location, patients who lived in Eastern Taiwan showed a significantly higher prevalence of scleritis, relative to those who lived in other geographic locations in Taiwan. Geographic location remained a significant risk factor after a conditional logistic regression analysis was conducted (Table 2). As for the residential city status of the patients residence, patients who lived in a rural area had a significantly higher prevalence of scleritis (OR  =  1.16, 95% confidence interval (CI)  =  1.13–1.18, *p*  <  0.0001), relative to those who lived in a metropolis city, and this remained a significant risk factor after a conditional logistic regression analysis was conducted (adjusted OR  =  1.15, 95% CI  =  1.12–1.18, *p*  <  0.0001) (Table 2). Patients whose occupation was as a public servant, including in the military, as civil, or as teaching staff, were at a significant risk of developing scleritis (OR  =  1.14, 95% CI  =  1.11–1.16, *p*  <  0.0001), which was still an independent risk factor after considering other confounders (adjusted OR  =  1.14, 95% CI  =  1.11–1.16, *p*  <  0.0001) (Table 2).

### 3.4. Associated Risk Factors—Comorbid Conditions

Several possible comorbidities were also examined using univariate and multiple logistic regression analyses (Table 2). The adjusted ORs from the multiple conditional logistic regression model were also adjusted for significant sociodemographic factors and comorbidities and conditioned on age group, sex, and the year of index date. Patients who had undergone ocular surgery, including pterygium removal, scleral flap creation during glaucoma surgery, and scleral buckle replacement had significantly higher ORs of receiving a diagnosis of scleritis (OR  =  3.98, 95% CI  =  3.65–4.35, *p*  <  0.0001; OR  =  2.70, 95% CI  =  2.00–3.63, *p*  <  0.0001; OR  =  6.68, 95% CI  =  5.32–8.39, *p*  <  0.0001, respectively), even after conditional logistic regression was conducted (adjusted OR  =  4.01, 95% CI  =  3.64–4.43, *p*  <  0.0001; adjusted OR  =  3.16, 95% CI  =  2.24–4.47, *p*  <  0.0001; adjusted OR  =  6.83, 95% CI  =  5.34–8.74, *p*  <  0.0001, respectively) (Table 2). Patients with a systemic infection, including syphilis, tuberculosis, or a human herpes viral infection, had significantly increased odds of a scleritis diagnosis before (OR  =  5.07, 95% CI  =  3.83–6.72, *p*  <  0.0001; OR  =  3.05, 95% CI  =  2.69–3.45, *p*  <  0.0001; OR  =  9.46, 95% CI  =  8.97–9.99, *p*  <  0.0001, respectively) and after adjustment for other confounders (adjusted OR  =  4.01, 95% CI  =  2.93–5.50, *p*  <  0.0001; adjusted OR  =  2.24, 95% CI  =  1.94–2.58, *p*  <  0.0001; adjusted OR  =  8.54, 95% CI  =  8.07–9.03, *p*  <  0.0001, respectively) (Table 2). Patients with systemic diseases, such as Sjögren syndrome, rheumatoid arthritis, granulomatous vasculitis, systemic lupus erythematosus, ankylosing spondylitis, and gout, had a significantly higher ORs of receiving a scleritis diagnosis (OR  =  44.93, 95% CI  =  36.93–54.67, *p*  <  0.0001; OR  =  5.18, 95% CI  =  4.76–5.64, *p*  <  0.0001; OR  =  13.67, 95% CI  =  7.61–24.56, *p*  <  0.0001; OR  =  6.53, 95% CI  =  5.51–7.74, *p*  <  0.0001; OR  =  6.92, 95% CI  =  6.26–7.65, *p*  <  0.0001; OR  =  3.23, 95% CI  =  3.11–3.36, *p*  <  0.0001, respectively), even after conditional logistic regression was conducted (adjusted OR  =  33.53, 95% CI  =  27.43–40.97, *p*  <  0.0001; adjusted OR  =  2.93, 95% CI  =  2.66–3.23, *p*  <  0.0001; adjusted OR  =  7.37, 95% CI  =  3.91–13.88, *p*  <  0.0001; adjusted OR  =  3.18, 95% CI  =  2.63–3.85, *p*  <  0.0001; adjusted OR  =  5.57, 95% CI  =  4.99–6.22, *p*  <  0.0001; adjusted OR  =  2.84, 95% CI  =  2.72–2.96, *p*  <  0.0001, respectively) (Table 2).

## 4. Discussion

To the best of our knowledge, this appears to be the largest nationwide, population-based case–control study to evaluate the association between sociodemographic factors, common comorbid conditions, and scleritis in Taiwan. Recently, Braithwaite et al. conducted a case–control and matched cohort study, which included 3005 scleritis patients, and found that, compared with controls, scleritis patients showed an increased risk of previous systemic immune-mediated inflammatory disease or an infectious condition [14]. Among conditions that are significantly associated with scleritis, diseases such as sjogren syndrome, rheumatoid arthritis, granulomatous vasculitis, systemic lupus erythematosus, ankylosing spondylitis, and human herpes viral infection were consistent with our report. Our analyses identified several key findings. First, more than half of the patients with scleritis in Taiwan were aged between 25 and 55 years old and the condition was more common in females, with a 56.84% predominance. Second, the odds of developing scleritis varied with several sociodemographic factors. Patients with an income > NT$ 30,000, living in either Eastern Taiwan or a rural area, and who were public servants, had higher odds of developing scleritis. Third, some comorbid conditions significantly influenced the odds of developing scleritis. By comparison, patients who had undergone ocular surgery, were in a systemic or local infectious state, or had a systemic autoimmune disease had significantly higher odds of developing scleritis. Of particular interest was that patients with Sjögren syndrome had considerably higher odds of developing scleritis compared to non-Sjögren syndrome subjects (adjusted OR  =  33.53, 95% CI  =  27.43–40.97, *p*  <  0.0001). 

We also studied the age and sex in scleritis patients. We found that of the 111,960 scleritis patients, 75,641 (67.56%) were aged between 2 and 55 years, and the average age at scleritis diagnosis was 42.92 years (SD 15.33). This finding was inconsistent with earlier investigations that reported scleritis affects patients in middle age, commonly between 47 and 60 years [1,3,4,5,6]. The inconsistency of the higher scleritis prevalence in young to middle age patients between our results and the previous reports may be related to racial differences, environmental factors, geographic location, and even perhaps reflecting differences in medical insurance systems, such as medical resources, medical information, and medical accessibility, which may affect the age distribution of the diagnoses of scleritis; such differences need to be clarified by more local epidemiologic scleritis studies in the future.

The patients with scleritis demonstrated a female preponderance in the current study, which is consistent with the female predominance previously reported [1,3,4,5,6]. We have attempted to explain why there was a higher prevalence of scleritis in women in the current study, based on the observation that females were more likely to have an autoimmune disease, which is an important etiology of scleritis development [15]. Whether sex hormones play a role in the pathophysiology of scleritis needs to be clarified with more research studies in the future.

Regarding the sociodemographic factors, we found statistically significant associations between scleritis and patients living in Eastern Taiwan, especially in rural areas. The higher rate of scleritis diagnoses in Eastern Taiwan and rural areas in our study may reflect those patients who live in these low economically developed areas, that may have poorer hygiene, and a higher possibility of exposure to mud or dirty water after ocular surgery, which might result in infection, and is an important risk factor for scleritis in the current study [7]. Another possible reason that explains the link between Eastern Taiwan, rural areas, and scleritis development is the unavailability of medical care, unease of visits to physicians, and lack of access to rheumatology specialists for diagnosis and management of autoimmune diseases, compared with other regions of Taiwan and other residential city statuses. The poorer the control of the autoimmune diseases, the higher the possibility of scleritis development. Individuals with an income >NT$ 30,000 or who were public servants had significantly higher odds of developing scleritis. This implies that patients with scleritis would not have limitations on employment due to their disease. We speculate that the risk of scleritis is influenced by income from multifactorial aspects. Income has an effect on lifestyle, living environment, and even psychological health. Moreover, previous studies have focused on factors such as age, sex, ethnicity, BMI, and smoking; however, they seldom discussed the role of income. Thus, we perfomed the trend test to relate income and the risk of scleritis in such patients. The trend test of estimated odds ratio for income and scleritis was *p* < 0.0001.

In the current study, approximately 3% of infectious scleritis diagnoses were associated with recent ocular surgery, with pterygium excision being the most common cause (Table 1). Recent pterygium surgery was indeed an independent risk factor of scleritis development (Table 2). This finding is consistent with previous reports, which showed that pterygium surgery was the most common procedure related to infectious scleritis [1,7]. Possible explanations for this finding include the technique of pterygium excision, excessive cauterization, and adjuvant topical antimetabolites, which have been implicated in the development of infectious scleritis [1,7]. Vascular tissue destruction during pterygium surgical manipulation likely results in increasing susceptibility to infection [8]. Regarding the association between ocular surgery and scleritis, our regression results show that scleral buckle replacement is the most important risk for scleritis (Table 2).

Additionally, we found that the herpes viruses are the most common pathogens associated with infectious scleritis and are involved in up to 12.53% of cases of scleritis. A recent human herpes viral infection appears to increase the risk of scleritis formation. Our finding is in accordance with several reports, whose results suggest that the most common infectious agents related to scleritis are the human herpes viruses [1,9]. Although the diagnosis of herpetic-associated scleritis may be challenging, several symptoms and signs that should raise suspicion include unilateral involvement, acute onset, moderate–intense pain, and associated uveitis or keratitis [1,9]. Prompt diagnosis and early treatment are crucial to reduce the risk of vision loss in herpetic-associated scleritis.

In the current study, patients with Sjögren syndrome had a remarkably high OR for scleritis development (adjusted OR  =  33.53, 95% CI  =  27.43–40.97, *p*  <  0.0001). Although the relationship between scleritis and systemic immune-mediated inflammatory conditions is well established [10,11], rheumatoid arthritis is considered the most common associated systemic disease, followed by systemic vasculitis diseases [12,13]. Sjögren syndrome, a common autoimmune disease, manifests as chronic and debilitating inflammation that is mediated by autoantibody production and lymphocytic infiltration [16]. This immune-mediated systemic inflammatory condition causes permanent destruction of exocrine glands, resulting in sicca symptoms, which are the most well-known and bothersome symptoms of Sjögren syndrome [16,17]. Of particular interest, we found that Sjögren syndrome was a prominent independent risk factor of scleritis formation. The association between Sjögren syndrome and scleritis has only been discussed in a few previous case studies [18,19,20], and our study appears to be the largest nationwide, population-based, case–control study to show this linkage. Recently, a case–control study that recruited 3005 scleritis patients showed that Sjögren syndrome is strongly associated with scleritis (adjusted OR  =  7.14, 95% CI  =  3.50–14.57, *p*  <  0.0001) [14]. Patients with undiagnosed Sjögren syndrome could also present with scleritis and symptoms other than dry eye; therefore, a timely referral for systemic workups is important for reducing delays in the diagnosis and improve the quality of life of patients with Sjögren syndrome. Meanwhile, the availability of effective treatment for scleritis, coupled with the high potential for vision loss due to this condition, demonstrates the need for a close collaboration between ophthalmologists and rheumatologists and familiarity with the various ocular manifestations of Sjögren syndrome.

In addition to immune-mediated systemic inflammatory conditions, we found that gout, a metabolite-mediated systemic inflammatory disease, also plays a role in scleritis development. Patients with gout had a significantly higher OR for scleritis development (adjusted OR  =  2.84, 95% CI  =  2.72–2.92, *p*  <  0.0001). An association between patients with gout and scleritis has been sparsely reported [21,22]. Gout, the most common type of inflammatory arthritis, results from an elevation in body uric acid levels, leading to deposition of monosodium urate crystals, mainly in the joints [23,24,25]. Gouty inflammation, which is associated with pro-inflammatory cytokines induced by exposure to monosodium urate crystals, could possibly contribute to scleritis formation in patients with gout.

Our study had several strengths. To the best of our knowledge, the current study is the largest to focus on patients with scleritis, and to show an association between scleritis and Sjögren syndrome. The selection bias resulting from referral centers was obviated, because the data was based on a nationwide and population-based dataset. As the data was obtained from the NHIRD database and the claims data for the NHIRD was recorded electronically rather than being reliant on the patient self-reporting their medical conditions, the recall bias was reduced. In addition, our study was case–control and incorporated 10 years of longitudinal data on various sociodemographic factors, post ocular surgery, systemic or ocular infectious diseases, and immune-mediated or metabolite-mediated systemic inflammation diseases in the patients with scleritis and controls. Our results are reliable, because the conditions mentioned above were recognized as potential confounding factors and underwent appropriate adjustments when assessing the OR in the patients with scleritis.

The current study had several limitations. First, the diagnosis of scleritis and other comorbid disorders were based on ICD-9-CM codes, which may lead to disease misclassification. In addition, the presence of scleritis in the patient group or the absence of scleritis in the control group based on the claims data could not be confirmed due to lack of access to clinical records. Second, the diagnosis of scleritis and other confounders were based on ICD-9-CM codes or order codes, which may result in misclassification. Finally, it could not be confirmed that the controls had not been diagnosed with scleritis before January 1996, because the medical history of the subjects could only be traced back to 1996. The current study only determined the more significantly associated factors in patients with scleritis and the general population, according to the study aims. The effects of sociodemographic and pathophysiologic factors should be further analyzed through future research using questionnaires and clinical information. Our study does not directly indicate causality, which should be evaluated in future studies.

## 5. Conclusions

In summary, the current study showed that living in Eastern Taiwan and residing in a rural area were associated with an increased risk of scleritis, but it appeared that patients with scleritis in Taiwan would not have employment or income limitations due to the disease. It is important to note that after controlling for sociodemographic factors and possible comorbidities, we found that patients with Sjögren syndrome had a significantly higher risk of developing scleritis than those with other well-known risk factors, such as post ocular surgery, systemic or ocular infectious diseases, and immune-mediated or metabolite-mediated systemic inflammation diseases.

## Figures and Tables

**Table 1:** Baseline sociodemographic factors and comorbid conditions of scleritis patients and control subjects after propensity score matching by age and sex.

	Scleritis *n* = 111,960	Control *n* = 111,960	*p* Value
Sociodemographic Factors	*n* (%)	*n* (%)	
Age (year; Mean ± SD)	42.92 ± 15.33	42.92 ± 15.33	0.9747 ^a^
Age (year)			
<25	12,080 (10.79)	12,080 (10.79)	1.0000 ^b^
25–34	26,700 (23.85)	26,700 (23.85)	
35–44	26,776 (23.92)	26,776 (23.92)	
45–54	22,165 (19.80)	22,165 (19.80)	
55–64	13,707 (12.24)	13,707 (12.24)	
≥65	10,532 (9.41)	10,532 (9.41)	
Gender			
Male	48,317 (43.16)	48,317 (43.16)	1.0000 ^b^
Female	63,643 (56.84)	63,643 (56.84)	
Income			
<NT$ 30,000	57,986 (51.79)	63,618 (56.82)	<0.0001 ^b^
NT$ 30,000–60,000	41,266 (36.86)	38,203 (34.12)	
NT$ 60,000–90,000	9458 (8.45)	7714 (6.89)	
NT$ 90,000–120,000	1725 (1.54)	1314 (1.17)	
>NT$ 120,000	1526 (1.36)	1111 (0.99)	
Geographical region of Taiwan			
Northern	61,637 (55.05)	56,835 (50.76)	<0.0001 ^b^
Central	13,787 (12.31)	21,263 (18.99)	
Southern	28,767 (25.69)	31,043 (27.73)	
Eastern	7769 (6.94)	2819 (2.52)	
Residential city status			
Metropolis	79,400 (70.92)	81,487 (72.78)	<0.0001 ^b^
Satellite	6996 (6.25)	7657 (6.84)	
Rural	25,564 (22.83)	22,816 (20.38)	
Occupation			
Public servant	71,669 (64.01)	66,402 (59.31)	<0.0001 ^b^
Farmer	7423 (6.63)	11,615 (10.37)	
Fisherman	1585 (1.42)	2193 (1.96)	
Other	31,283 (27.94)	31,750 (28.36)	
Comorbid conditions			
Post Surgery			
Post-pterygium removal	2513 (2.24)	659 (0.59)	<0.0001 ^b^
Post-glaucoma surgery	159 (0.14)	59 (0.05)	<0.0001 ^b^
Post-scleral buckle replacement	568 (0.51)	85 (0.08)	<0.0001 ^b^
Infection			
Syphilis	294 (0.26)	58 (0.05)	<0.0001 ^b^
Tuberculosis	1005 (0.90)	334 (0.30)	<0.0001 ^b^
Human herpesvirus infection	14,026 (12.53)	1669 (1.49)	<0.0001 ^b^
Systemic Disease			
Sjögren syndrome	4588 (4.10)	107 (0.10)	<0.0001 ^b^
Rheumatoid arthritis	3298 (2.95)	658 (0.59)	<0.0001 ^b^
Granulomatous vasculitis	164 (0.15)	12 (0.01)	<0.0001 ^b^
Systmic lupus erythematosus	1009 (0.90)	157 (0.14)	<0.0001 ^b^
Ankylosing spondylitis	3012 (2.69)	449 (0.40)	<0.0001 ^b^
Gout	11,313 (10.10)	3952 (3.53)	<0.0001 ^b^

^a^ Paired *t*-test; ^b^ McNemar’s test; NT$, New Taiwan dollars; SD, standard deviation.

**Table 2:** Odds ratios and adjusted odds ratios for various sociodemographic factors and comorbid conditions with scleritis.

	Odds Ratio ^a^ (95% CI)	*p* Value	Adjusted Odds Ratio ^b^ (95% CI)	*p* Value
Sociodemographic factors				
Income				
<NT$ 30,000	1.00		1.00	
NT$ 30,000–60,000	1.22 (1.19–1.24)	<0.0001	1.19 (1.16–1.21)	<0.0001
NT$ 60,000–90,000	1.40 (1.36–1.45)	<0.0001	1.36 (1.31–1.41)	<0.0001
NT$ 90,000–120,000	1.51 (1.40–1.62)	<0.0001	1.46 (1.35–1.59)	<0.0001
>NT$ 120,000	1.57 (1.46–1.70)	<0.0001	1.55 (1.42–1.69)	<0.0001
Geographical region of Taiwan				
Northern	0.39 (0.38–0.41)	<0.0001	0.42 (0.40–0.44)	<0.0001
Central	0.24 (0.22–0.25)	<0.0001	0.24 (0.23–0.26)	<0.0001
Southern	0.34 (0.32–0.35)	<0.0001	0.37 (0.35–0.39)	<0.0001
Eastern	1.00		1.00	
Residential city status				
Metropolis	1.00		1.00	
Satellite	0.94 (0.91–0.97)	0.0002	0.92 (0.89–0.96)	<0.0001
Rural	1.16 (1.13–1.18)	<0.0001	1.15 (1.12–1.18)	<0.0001
Occupation				
Public servant	1.14 (1.11–1.16)	<0.0001	1.14 (1.11–1.16)	<0.0001
Farmer	0.60 (0.58–0.62)	<0.0001	0.53 (0.51–0.55)	<0.0001
Fisherman	0.75 (0.70–0.80)	<0.0001	0.66 (0.61–0.71)	<0.0001
Other	1.00		1.00	
Comorbid conditions				
Post Surgery				
Post-pterygium removal	3.98 (3.65–4.35)	<0.0001	4.01 (3.64–4.43)	<0.0001
Post-glaucoma surgery	2.70 (2.00–3.63)	<0.0001	3.16 (2.24–4.47)	<0.0001
Post-scleral buckle replacement	6.68 (5.32–8.39)	<0.0001	6.83 (5.34–8.74)	<0.0001
Infection				
Syphilis	5.07 (3.83–6.72)	<0.0001	4.01 (2.93–5.50)	<0.0001
Tuberculosis	3.05 (2.69–3.45)	<0.0001	2.24 (1.94–2.58)	<0.0001
Human herpes viral infection	9.46 (8.97–9.99)	<0.0001	8.54 (8.07–9.03)	<0.0001
Systemic Disease	13.67 (7.61–24.56)	<0.0001	7.37 (3.91–13.88)	<0.0001
Sjögren syndrome	44.93 (36.93–54.67)	<0.0001	33.53 (27.43–40.97)	<0.0001
Rheumatoid arthritis	5.18 (4.76–5.64)	<0.0001	2.93 (2.66–3.23)	<0.0001
Granulomatous vasculitis	13.67 (7.61–24.56)	<0.0001	7.37 (3.91–13.88)	<0.0001
Systmic lupus erythematosus	6.53 (5.51–7.74)	<0.0001	3.18 (2.63–3.85)	<0.0001
Ankylosing spondylitis	6.92 (6.26–7.65)	<0.0001	5.57 (4.99–6.22)	<0.0001
Gout	3.23 (3.11–3.36)	<0.0001	2.84 (2.72–2.96)	<0.0001

^a^ Odds ratios (ORs) were obtained from a univariate logistic regression analysis; ^b^ adjusted ORs were calculated using a multivariable conditional logistic regression model with significant univariate ORs (*p* < 0.05), and this model was conditioned on age group, sex, and the year of index date. NT$, New Taiwan dollars; CI, confidence interval.

## Data Availability

All relevant data are within the paper.
